# Co-aggregation of pro-inflammatory S100A9 with α-synuclein in Parkinson’s disease: ex vivo and in vitro studies

**DOI:** 10.1186/s12974-018-1210-9

**Published:** 2018-06-04

**Authors:** Istvan Horvath, Igor A. Iashchishyn, Roman A. Moskalenko, Chao Wang, Sebastian K. T. S. Wärmländer, Cecilia Wallin, Astrid Gräslund, Gabor G. Kovacs, Ludmilla A. Morozova-Roche

**Affiliations:** 1 Department of Medical Biochemistry and Biophysics, Umeå University, 90187 Umeå, Sweden; 2 Department of General Chemistry, Sumy State University, Sumy, 40007 Ukraine; 3 Department of Pathology, Sumy State University, Sumy, 40007 Ukraine; 4 Department of Biochemistry and Biophysics, Stockholm University, 10691 Stockholm, Sweden; 5 Institute of Neurology, Medical University of Vienna, Vienna, Austria

**Keywords:** S100A9, α-Synuclein, Parkinson’s disease, Neuroinflammation, Amyloid, Cytotoxicity

## Abstract

**Background:**

Chronic neuroinflammation is a hallmark of Parkinson’s disease (PD) pathophysiology, associated with increased levels of pro-inflammatory factors in PD brain tissues. The pro-inflammatory mediator and highly amyloidogenic protein S100A9 is involved in the amyloid-neuroinflammatory cascade in Alzheimer’s disease. This is the first report on the co-aggregation of α-synuclein (α-syn) and S100A9 both in vitro and ex vivo in PD brain.

**Methods:**

Single and sequential immunohistochemistry, immunofluorescence, scanning electron and atomic force (AFM) microscopies were used to analyze the ex vivo PD brain tissues for S100A9 and α-syn location and aggregation. In vitro studies revealing S100A9 and α-syn interaction and co-aggregation were conducted by NMR, circular dichroism, Thioflavin-T fluorescence, AFM, and surface plasmon resonance methods.

**Results:**

Co-localized and co-aggregated S100A9 and α-syn were found in 20% Lewy bodies and 77% neuronal cells in the substantia nigra; both proteins were also observed in Lewy bodies in PD frontal lobe (Braak stages 4–6). Lewy bodies were characterized by ca. 10–23 μm outer diameter, with S100A9 and α-syn being co-localized in the same lamellar structures. S100A9 was also detected in neurons and blood vessels of the aged patients without PD, but in much lesser extent. In vitro S100A9 and α-syn were shown to interact with each other via the α-syn C-terminus with an apparent dissociation constant of ca. 5 μM. Their co-aggregation occurred significantly faster and led to formation of larger amyloid aggregates than the self-assembly of individual proteins. S100A9 amyloid oligomers were more toxic than those of α-syn, while co-aggregation of both proteins mitigated the cytotoxicity of S100A9 oligomers.

**Conclusions:**

We suggest that sustained neuroinflammation promoting the spread of amyloidogenic S100A9 in the brain tissues may trigger the amyloid cascade involving α-syn and S100A9 and leading to PD, similar to the effect of S100A9 and Aβ co-aggregation in Alzheimer’s disease. The finding of S100A9 involvement in PD may open a new avenue for therapeutic interventions targeting S100A9 and preventing its amyloid self-assembly in affected brain tissues.

**Electronic supplementary material:**

The online version of this article (10.1186/s12974-018-1210-9) contains supplementary material, which is available to authorized users.

## Background

PD is the most common age-dependent neurodegenerative movement disorder affecting about 2% of the population over 60 years old. The hallmark of PD is pathological self-assembly of the amyloidogenic protein α-synuclein (α-syn), which forms cytotoxic amyloid oligomers and mature fibrils in PD brain tissues [1, 2]. Native α-syn exists as an intrinsically disordered monomer. In the presence of lipid membranes, α-syn undergoes a conformational change to a folded α-helical secondary structure [3]. Although the biological functions of α-syn are debated, it has been suggested to play role in maintaining a supply of synaptic vesicles in presynaptic terminals by clustering synaptic vesicles, and to be involved in regulating the release of the neurotransmitter dopamine in controlling voluntary and involuntary movements [2, 4]. Despite numerous studies, the critical factors triggering α-syn aberrant conversion into pathological β-sheet-rich amyloid aggregates and consequently initiation of PD remain unclear. The majority of PD incidents are sporadic, but inherited α-syn mutations leading to its amyloid formation at early onset are present in familial PD, which constitute ca. 10–15% of all PD cases. When α-syn assembles into amyloid fibrils, these aggregates accumulate in the form of Lewy bodies and Lewy neurite deposits in neuronal cells primarily in the substantia nigra. These deposits are also found in the frontal lobe, vagus dorsal motor nuclei, nucleus basalis of Meynert, and locus coeruleus [5–7]. Amyloid oligomers of α-syn are considered to be a major cause of neuronal cell toxicity and progressive neurodegeneration [8]. The latter leads to PD pathology, which manifests in the loss of motor function, bradykinesia, rigidity, instability, and tremor. Apart from the pathogenic neurotoxicity of oligomeric α-syn, the depletion of α-syn monomers in their physiological locations due to their aggregation may also contribute to neurodegeneration [3]. An interesting idea of prion-like transmission of α-syn pathology between neuronal cells has recently been proposed [9], highlighting that α-syn in an aberrantly folded, β-sheet-rich conformation can migrate from affected to unaffected neurons, and thus trigger amyloid templating in the host cells. This indicates that once aggregation begins, it can effectively spread to the surrounding tissues, leading to PD progression.

Growing evidence has been accumulated concerning the importance of additional causal factors that can contribute to, or turn on, the pathological cascade of α-syn amyloid aggregation. The most obvious common denominator in major neurodegenerative diseases, including PD and Alzheimer’s disease, is neuroinflammation [10, 11]. Previously, neuroinflammation was simply regarded as a response to neurodegeneration in these diseases. However, recent studies suggest that neuroinflammation could be the trigger and the key player in neurodegenerative diseases by creating a pathogenic microenvironment in the brain tissues. PD brains showed extensive microglial activation, infiltration of blood-derived mononuclear phagocytes and lymphocytes, and significant rise of pro-inflammatory cytokines—all deleterious responses, which can sustain inflammation and exacerbate neurodegeneration [11–13]. Moreover, epidemiological studies have demonstrated that nonsteroidal anti-inflammatory drugs may lower the risk of neurodegenerative diseases, including both Alzheimer’s disease and PD [12, 14].

The importance of co-aggregation of amyloidogenic proteins in a number of neurodegenerative diseases has recently been highlighted [15]. It has been shown that molecules designed to inhibit aggregation of one amyloidogenic protein may inhibit the aggregation of others [16, 17] and thus potentially affect the whole amyloid cascade.

In this study, we have focused on the involvement in PD pathology of the specific pro-inflammatory mediator S100A9, which we have found to play a critical role in connecting neuroinflammatory and amyloid pathologies into the integrated amyloid-neuroinflammatory cascade in Alzheimer’s disease [18]. S100A9 belongs to the family of structurally homologous calcium-binding S100 proteins, which are broadly involved in many inflammatory, cancer, and neurodegenerative conditions [19]. We have previously shown that S100A9 is highly amyloidogenic and easily forms amyloid oligomers and fibrils under in vitro conditions that mimic physiological conditions [18, 20]. In Alzheimer’s disease, S100A9 was found to be abundant both in neuronal cells and in amyloid plaques, prompting co-aggregation with amyloid-β (Aβ), the major amyloidogenic peptide in Alzheimer’s disease [18]. It has therefore been suggested that pro-inflammatory S100A9, which possesses intrinsic amyloidogenic properties as well as the ability to modulate Aβ aggregation, can serve as a link between the Alzheimer’s disease amyloid and neuroinflammatory cascades and as a prospective therapeutic target [18]. Interestingly, the critical role of S100A9 in Alzheimer’s disease development was demonstrated in a mouse model, where S100A9 production was induced by both the Aβ peptide and the C-terminal fragment of the Aβ precursor protein, while S100A9 knockdown attenuated memory impairment and reduced amyloid plaque burden [21]. Among the brain pathologies, a widespread expression of S100A9 has been reported in cerebral malaria [22], cerebral ischemia [23], Alzheimer’s disease [18, 24], and traumatic brain injury [25], where it may initiate sustainable inflammatory responses and perform mediator functions controlling inflammatory responses of other cells. An abundance of S100A9 mRNA has recently been identified as a strong feature of aging in various mammalian tissues, including the central nervous system, and a novel mechanism of age-associated inflammation sustained by S100A9 has been suggested [26].

The role of S100A9 in PD remains to be elucidated. There are clear indications that another protein from the S100 protein family—S100B—plays a role in PD [27, 28]. Elevated S100B levels were found in the postmortem substantia nigra of PD patients compared with control tissues, and S100B levels in the cerebrospinal fluid were also higher in a large cohort of PD patients compared with controls [27]. Autoimmune responses to S100B in the blood sera of PD patients were also significantly higher than in control subjects [28]. Moreover, transgenic mice overexpressing S100B developed PD features, resulting in motor coordination impairment [29].

Here, by using combined analysis of ex vivo PD-affected brain tissues and S100A9 co-aggregation with α-syn in vitro, we show that S100A9 indeed complements α-syn amyloid aggregation and is involved in the amyloid-neuroinflammatory cascade in PD pathology.

## Methods

### Proteins

S100A9 (13.2 kDa, 114 amino acid residues) and α-syn (14.4 kDa, 140 residues) were expressed and purified from *E*. *coli* as described previously [30, 31]. Proteins were freeze-dried and used directly after dissolving them in phosphate buffered saline (PBS, 09-8912-100, Medicago). All samples were passed through a 0.22-μm filter to eliminate spontaneously formed aggregates. ^15^N-labeled α-syn was purchased from AlexoTech AB. Protein concentrations were determined by absorption at 280 nm with extinction coefficients of *ε*_280_ = 0.53 (mg/mL)^−1^ cm^−1^ for S100A9 and 0.41 (mg/mL)^−1^ cm^−1^ for α-synuclein, respectively.

### Tissue samples

The postmortem brain tissues from five PD patients and four controls were examined (Table 1). Controls had no neurodegenerative diseases, in particular no Lewy bodies or neurofibrillary tangles or anything else indicative of a neurodegenerative condition in the substantia nigra or frontal lobe as confirmed by the neuropathologist collaborator at the Institute of Neurology, Medical University of Vienna, Vienna, Austria, who provided the tissue samples for analysis. All tissue sections were from the frontal lobe and midbrain regions. They were paraffin-embedded and microtome-sectioned to 5-μm-thick slices.

**Table 1:** Characteristics of PD and control subjects, including age, gender, and stage of PD [49]

	Age	Gender	Stage
PD patients	83	Female	Braak 5
	69	Male	Braak 5
	83	Male	Braak 5
	82	Male	Braak 6
	75	Male	Braak 4
Control patients	83	Female	–
	69	Male	–
	83	Male	–
	75	Male	–

### Immunohistochemistry

Single and sequential immunohistochemistry with a series of antibodies applied to the same tissue sections were performed as described previously [32] with some modifications [33]. The following primary antibodies from Santa Cruz Biotechnology were used: S100A9 (rabbit polyclonal, sc-20173, 1 in 100), α-syn (mouse monoclonal, 3G282: sc-69977, 1 in 100, raised against recombinant α-syn of human origin), and CD68 (mouse monoclonal, sc70761, 1 in 100). In some control experiments, α-syn mouse monoclonal, LB 509 (Novakemi AB), was also used, raised against Lewy bodies purified from patients suffering dementia with Lewy bodies, and reactive with α-syn epitope located in the region of amino acids 115-122. Both α-syn antibodies showed consistent immunostaining. Secondary antibodies from Vector Laboratories were used: anti-mouse (MP-7402) and anti-rabbit (MP-7401) IgG peroxidase reagent kits. 3,3′-Diaminobenzidine (DAB) and 3-amino-9-ethylcarbazole (AEC) from Vector Laboratories were used as substrates producing brown and reddish-brown staining of the samples, respectively. The tissues were scanned by a Panoramic SCAN slide scanner 250 (3D Histech). Quantifications of S100A9-immunopositive cells in the substantia nigra and frontal lobe areas of five PD patients and four controls were conducted by selecting six random areas of 1 × 1 mm^2^ size in each tissue section and calculating the mean value over all counts. The percentage of co-localization of S100A9 and α-syn within cells in the substantia nigra was counted relative to α-syn-immunopositive cells. Lewy bodies were counted in the whole substantia nigra and frontal lobe regions of five PD patients, and co-localization of S100A9 and α-syn was calculated relative to α-syn-immunopositive inclusions.

### Immunofluorescence

The brain tissues were cut into 5-μm-thick sections, deparaffinized in xylene, rehydrated in a graded series of alcohol, followed by antigen retrieval in citrate buffer, pH 6.0, and washed in Tris-buffered saline Tween. The tissue sections were blocked with 5% bovine serum albumin (BSA) in PBS for 30 min at 37 °C, and then incubated during 1 h with primary antibodies diluted in 2.5% BSA. The following primary antibodies from Santa Cruz Biotechnology were used: α-syn (mouse monoclonal, sc-69977, 1 in 200) and S100A9 (rabbit polyclonal, sc-20173, 1 in 200). Then, the tissue samples were washed twice in PBS and incubated with Alexa Fluor 555 goat anti-mouse IgG secondary antibodies (10 μg/mL, A28180, Thermo Fisher Scientific), interacting with α-syn-specific primary antibodies, and observed by orange fluorescence and with Alexa Fluor 488 goat anti-rabbit IgG secondary antibodies (10 μg/mL, A-11034, Thermo Fisher Scientific), interacting with S100A9-specific primary antibodies, and observed by green fluorescence. The secondary antibodies were diluted in 2.5% mouse and rabbit serum, respectively; they were applied for 30 min at room temperature. Cell nuclei were stained with 4′,6-Diamidine-2′-phenylindole dihydrochloride (DAPI, Roche). Fluorescence microscopy was conducted on an Axio Imager A1 microscope (Carl Zeiss).

### Congo red staining

Deparaffinized and rehydrated tissue sections of 5 μm thickness were stained with a saturated ethanol solution of Congo red (Sigma) and sodium chloride and taken to pH 10.0 with 1% sodium hydroxide. Cell nuclei were stained with hematoxylin (Vector Laboratories). The stained samples were examined in an optical microscope (Leica DM LB).

### Amyloid formation kinetics

To produce amyloid aggregates, S100A9 and α-syn proteins were incubated in PBS, pH 7.4 and 37 °C. The amyloid formation kinetics was monitored as described previously [20]. To monitor amyloid formation kinetics, 20 μM thioflavin T (ThT) was initially added to native protein samples. The samples were placed in 96-well non-binding black plates (Corning), subjected to agitation at 300 rpm, and ThT fluorescence intensity was monitored by a Tecan Infinite F200 plate reader.

S100A9 and α-syn were cross-seeded with 5 and 10% of pre-formed amyloids of their counter-parts (molar percentage). The α-syn/S100A9 amyloid cross-seeding experiments were performed at the above conditions under 200 rpm agitation and their ThT fluorescence was recorded by a Fluostar Optima (BMG Labtech) plate reader. Fluorescence excitation wave length was set at 440 nm, and emission was registered at 480 nm every 10 min. Each sample was measured at least in eight replicates.

### Amyloid kinetic fitting

Fitting of the amyloid kinetic curves was performed by using a sigmoidal function described in [34]

1 $$ I(t)={I}_{\mathrm{min}}+\frac{I_{\mathrm{max}}}{1+{e}^{-\frac{t-{t}_0}{\tau }}}, $$

where *I*(*t*) is normalized fluorescence intensity, *I*_min_—fluorescence intensity at time 0, *I*_max_—fluorescence intensity at the plateau level, *t*_0_—midpoint of the growth phase (inflection point), *τ*—characteristic reaction time equal to 1*/K*_app_, where *K*_app_ is an apparent reaction rate constant. Lag-phase time is defined as *t*_lag_ *= t*_0_*−*2*τ*. Fitting was conducted by using a Wolfram Mathematica 11 package. Each experimental amyloid formation kinetic curve is an average of five repeats, and each repeat was fitted by Eq. (1). The rates derived from all repeats were subjected to statistical analysis to determine if the difference between the group rates is statistically significant. Level of statistical significance was set at 0.05.

### AFM

AFM imaging of the protein fibrils and tissue samples was carried out by a BioScope Catalyst AFM (Bruker) in peak force mode in air, with resolution of 512 × 512 pixels. ScanAsyst air cantilevers (Bruker) were used. Protein samples were diluted 50 times in deionized water and incubated on the surface of freshly cleaved mica for 15 min, washed three times with 100 μl deionized water, and dried at room temperature. Instrumental set up including a BioScope Catalyst AFM combined with an inverted Nicon-Ti-S microscope equipped with tissue slide holder was used for combining the immunohistochemical staining pattern with AFM imaging in analysis of Lewy bodies in the substantia nigra brain tissues.

### Lewy body dimensions

**Fig. 1:**
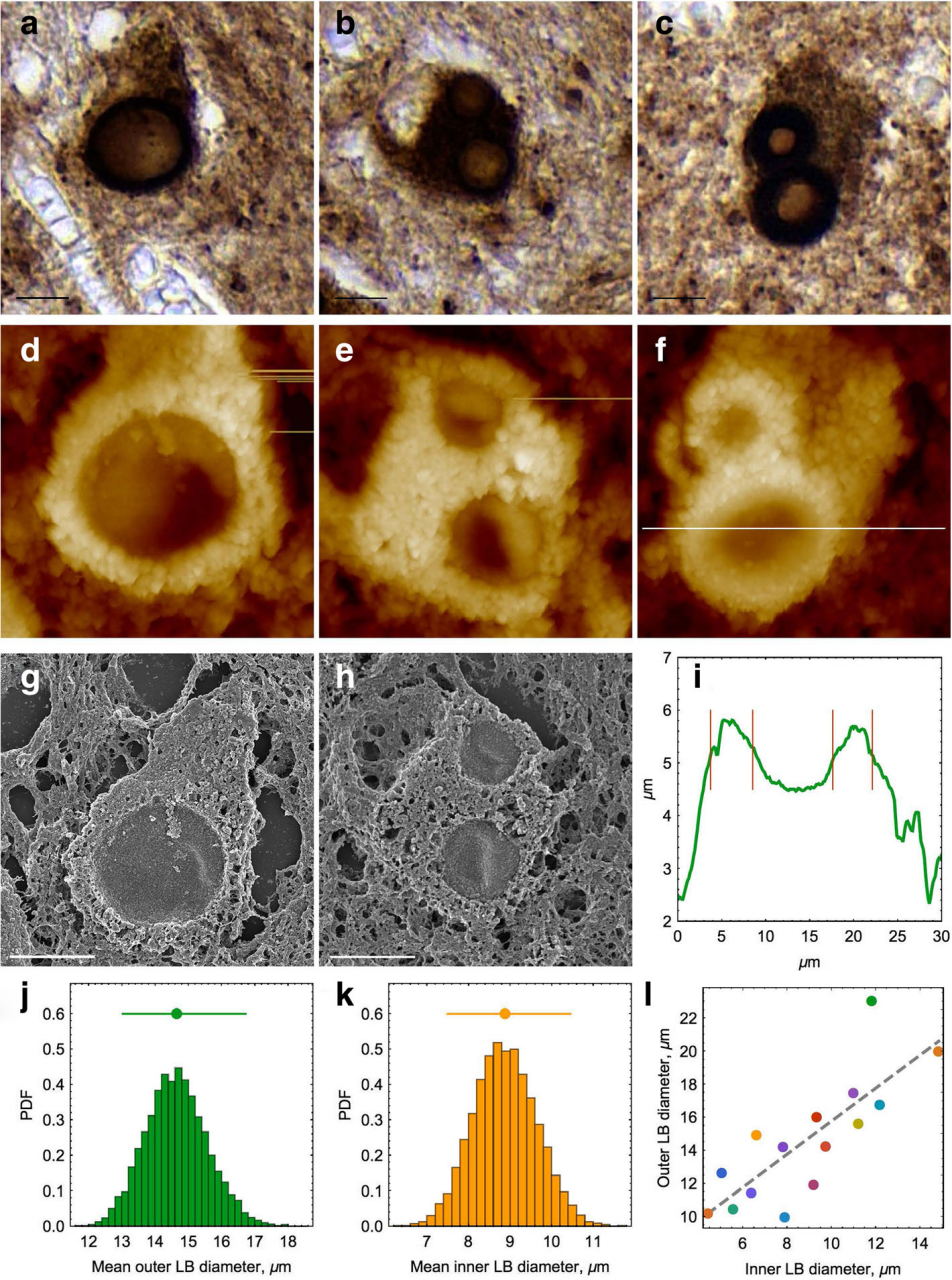
Microscopy of α-syn Lewy bodies in the PD substantia nigra (an individual case). **a**–**c** Representative intracellular Lewy bodies immunostained with α-syn antibodies. Lewy bodies are shown in dark brown color and the host cells in lighter brown shade. Scale bars equal to 10 μm. **d**–**f** AFM height images of the corresponding Lewy bodies (from **a**–**c**). The surfaces of Lewy bodies and surrounding tissues are covered with DAB crystals used in immunohistochemical procedure to stain the tissue samples (shown in light color). Image sizes are 20 × 20 μm. **g**, **h** Scanning electron microscopy images of Lewy bodies shown in **a**, **b**. **i** AFM cross-section of Lewy body; its position is shown in **f** by white line. **j**, **k** Distribution of Lewy body mean outer and inner diameters, respectively, calculated by using BCa technique from AFM data. Mean diameters and their 95% CI are shown above the histograms. Probability density function (PDF) is shown along the *y*-axis. **l** Linear dependence between the inner and outer diameters of the Lewy bodies analyzed by AFM. Each point represents individual randomly selected Lewy body from the same patient and is shown in individual color

Lewy body dimensions from a single patient were measured by using AFM topographic imaging, which provides higher accuracy compared to immunohistochemical staining. The measurements were performed in the AFM cross-sections of topographic images as shown in Fig. 1i. The estimates of outer and inner diameters of Lewy body toroidal structures are shown by red lines, drawn through the inflection points in their cross-sections, which were determined by first derivatives of the corresponding cross-section profiles.

### Scanning electron microscopy

Scanning electron microscopy of the brain tissue samples was performed by a Carl Zeiss Merlin field emission scanning electron microscope using accelerating voltage of 4 kV. Prior to imaging process, the tissue slide was coated with carbon in a Quorum Q150T-ES specimen preparation station.

### Cellular toxicity

SH-SY5Y neuroblastoma cells were cultured as described previously [35]. Cells were plated at a density of ca. 100 cells/well in 96-well plates; the medium was changed after 24 h of incubation and before adding protein samples. Initially, S100A9 and α-syn were incubated at concentrations of 70 μM in PBS, pH 7.4, and 37 °C during 10 and 60 h in order to produce the amyloid samples populated with oligomers/protofilaments and fibrils, respectively. The time points of sample collection were selected in accord with the kinetics of amyloid formation and AFM imaging for all protein specimens. Freshly dissolved and pre-incubated amyloid samples of S100A9, α-syn, and their mixture were diluted in the culture medium and added to SH-SY5Y cells at a final concentration of 10 μM. Cell viability was measured by WST-1 assay (Roche) after 24 h of co-incubation with added protein samples. Absorbance at 450 nm was measured in a plate reader (Tecan Infinite F200). Cell viability was expressed as a percentage of the absorbance in wells containing control cells with added PBS. All experiments were performed at least in triplicates, and each series was repeated three times. The amyloid samples were incubated in sterile conditions prior to adding to the cell culture media.

### Circular dichroism (CD)

Far UV CD spectra of both S100A9 and α-syn were recorded in 10 mM phosphate buffer, pH 7.4, and 20 °C with a Jasco J-710 spectropolarimeter using a 1-mm path length quartz cuvette.

### NMR spectroscopy

A Bruker Avance 700 MHz NMR spectrometer equipped with a triple-resonance cryoprobe was used to perform NMR measurements. 2D ^1^H,^15^N heteronuclear single quantum coherence (HSQC) spectra of 77 μM isotope-labeled α-syn in 10 mM phosphate buffer, pH 7.35, and 10 °C were recorded before and after addition of 2.5 mM S100A9. The spectra were referenced to the water signal, and the assignment of α-syn amide cross-peaks was used from previous work [36].

### Surface plasmon resonance

The interaction between α-syn and S100A9 were examined by using a Biacore X100 surface plasmon resonance instrument (GE Healthcare). Monomeric α-syn was immobilized on a streptavidin-coated chip as described previously [37]. The binding was measured in HBS-P+ buffer containing 10 mM HEPES, 150 mM NaCl, and 0.001% P20 detergent at pH 7.4 (GE Healthcare) and 25 °C. Increasing concentrations of S100A9 were injected in a single cycle sequence without regeneration steps between injections. At the end of the cycle, bound protein was removed by injection of 50 mM NaOH. Experiments were repeated three times. Dissociation constant *K*_d_ was evaluated with the program provided by the instrument manufacturer using the equation: *R*_c_ = (C*R_max_)/(*K*_d_ + *C*), where *R*_c_ denotes binding level at concentration *C* and *R*_max_ is the extrapolated maximum binding capacity.

### Statistical analysis

The normality of all data sets was assessed by the Shapiro-Wilk test. Values of *p* ≤ 0.05 were considered statistically significant. The experimental data sets were normally distributed and therefore analyzed by using two-sample *T* test. These results are shown as mean ± standard deviation (SD).

The means of outer and inner diameters of Lewy bodies (13 bodies) and their 95% confidence intervals (CI 95%) were calculated by corrected and accelerated bootstrap (BCa) technique [38, 39].

## Results

### S100A9 and α-syn in Lewy bodies in the PD substantia nigra and frontal lobe regions

The tissue samples from five PD patients and four control individuals (Table 1) were subjected to immunohistochemical analysis to examine the localization of α-syn and S100A9 antigens. Since Lewy body formation in the substantia nigra is a hallmark of PD pathology [40], we have examined the prevalence of intracytoplasmic Lewy bodies reactive with α-syn antibodies in the substantia nigra of five PD patients. A large number of Lewy bodies distributed all over the substantia nigra were detected in all PD patients, and in one representative patient, they were studied in more detail by combining immunohistochemistry and AFM imaging. Lewy bodies were strongly immunoreactive with α-syn antibodies as shown in the representative images in Fig. 1a–c, displaying characteristic pattern with a bright ring-shaped staining around the pale central core. Most Lewy bodies were located within neuronal cells shown in lighter brown shade at their background. Some neuronal cells contained two Lewy bodies (Fig. 1b, c), which is typical for PD pathology. This indicates that once the process of amyloid self-assembly has started within a cell, the developed amyloids can seed and propagate themselves.

The topographic AFM images of the same Lewy bodies in the substantia nigra tissues are shown in Fig. 1d–f, the images were scanned by positioning the AFM cantilever over the optical images of corresponding Lewy bodies. Since the Lewy bodies were initially localized within the brain sections by immunostaining, the surfaces of their sections were covered by DAB crystals used in immunohistochemical procedure. These surfaces are higher and displayed in a light color in AFM images, while the central parts not reactive with α-syn antibodies are shown in darker color, respectively (Fig. 1d–f). It was suggested that a granular core of Lewy bodies may include a variety of nitrated, phosphorylated, and ubiquitinated proteins surrounded by a filamentous halo containing α-syn amyloid fibrils [40]. The same individual Lewy bodies were imaged also by using scanning electron microscopy as shown in Fig. 1g, h, where they display the same morphology. Since the immunopositive parts of Lewy bodies are visible as annuli, we measured their outer and inner diameters in the AFM cross-sections (Fig. 1f, i). By using corrected and accelerated bootstrap technique, we calculated the probability density functions for means of both Lewy body diameters and their respective 95% confidence intervals (Fig. 1j, k). The mean value for outer diameters of all examined Lewy bodies was 14.7 μm (CI 95% 13.0–16.7) and for the inner diameters 7.9 μm (CI 95% 8.5–10.4), respectively. The dependence between the outer and inner diameters of Lewy bodies is linear with a slope of 0.99, indicating that the thickness of the annuli is proportional to their diameters (Fig. 1l). The diameters of Lewy bodies were also measured by using scanning electron microscopy images (Fig. 1g, h), which resulted in the dimensions consistent with AFM measurements.

**Fig. 2:**
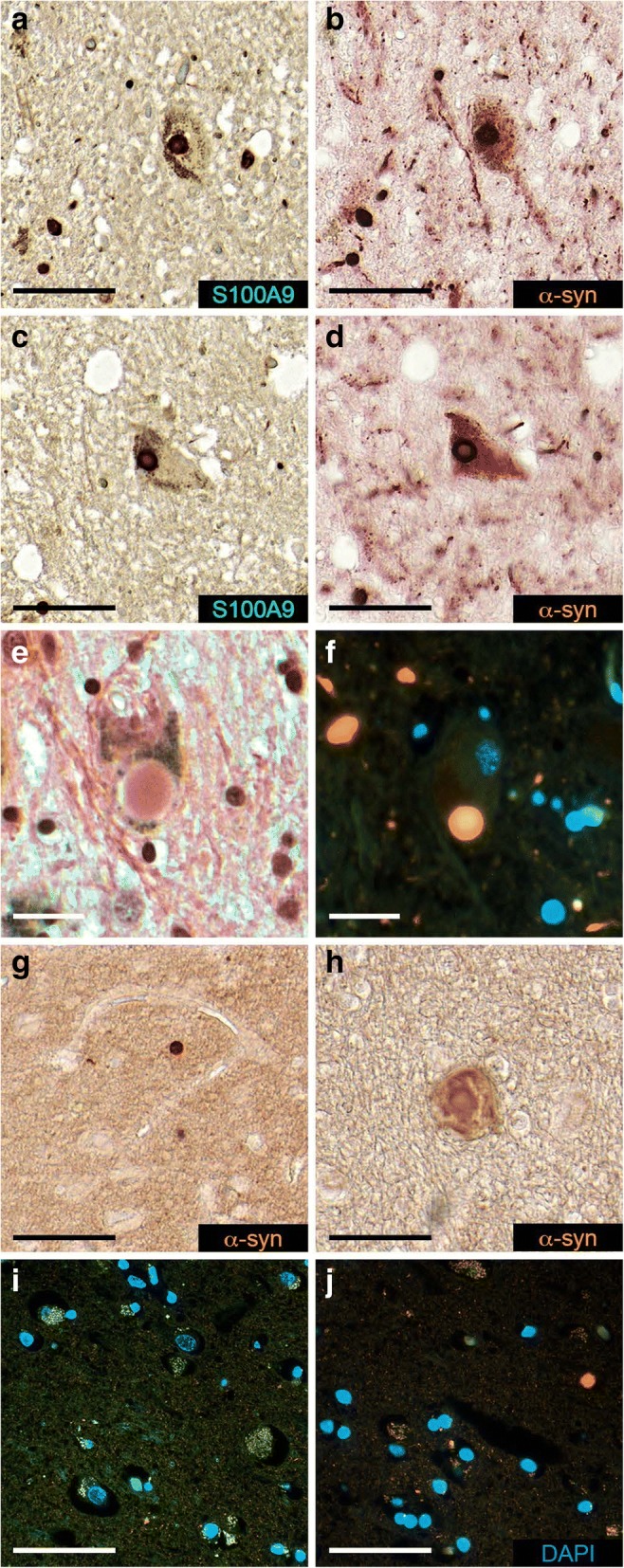
Optical and fluorescence microscopy of Lewy bodies in the substantia nigra and frontal lobe regions of five PD patients. Representative sequential immunohistochemistry of Lewy bodies formed in neuronal cells in the PD substantia nigra tissues and conducted by using the pair of consecutive S100A9 (**a**, **c**) and α-syn (**b**, **d**) antibodies, respectively. **e** Congo red staining of intracellular Lewy body in the substantia nigra. **f** Immunofluorescence of the representative substantia nigra tissue with α-syn antibodies (shown in orange), S100A9 antibodies (in green), their superposition (in yellow), and DAPI staining of cell nuclei (in blue). The Lewy body in the center of image is shown as a well-defined round-shaped intracellular inclusion. The Lewy body in the lower right corner is shown in yellow indicating staining with both S100A9 and α-syn antibodies. **g**, **h** Immunohistochemistry of the representative frontal lobe tissues with α-syn antibodies. **i**, **j** Immunofluorescence of the representative frontal lobe tissues with α-syn and S100A9 antibodies and DAPI staining. Color representation as in **f**. In the images, the labelling notes for S100A9 and α-syn antibody staining is shown in green and orange, respectively. Scale bars are 50 μm in **a**–**d**, **g**, and **h**, 20 μm in **e**, **f**, and 100 μm in **i**, **j**

**Fig. 3:**
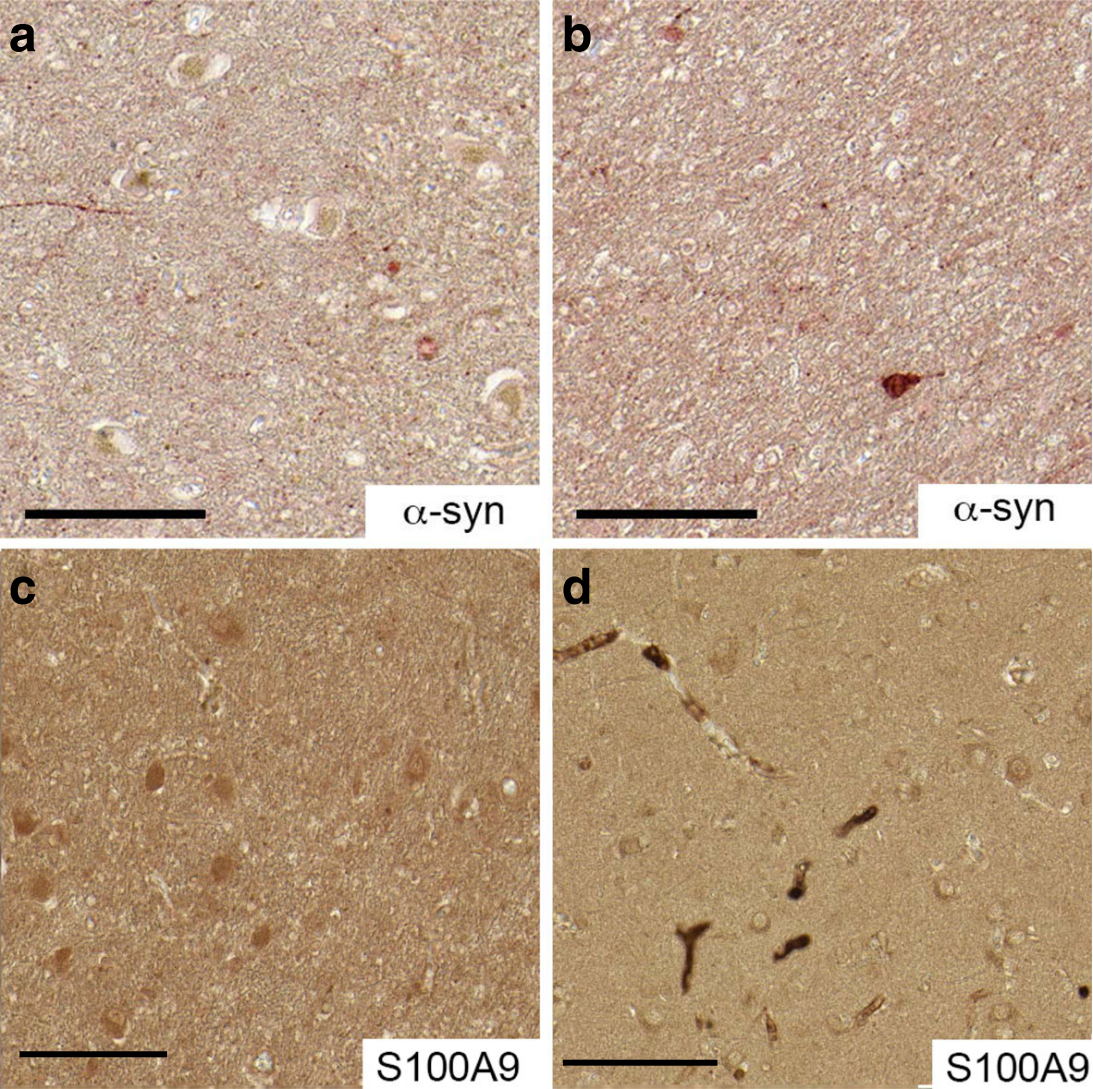
Immunohistochemical analysis of the brain tissues of four control cases. Representative immunostaining with α-syn (**a**, **b**) and S100A9 (**c**, **d**) antibodies of the substantia nigra (**a**, **c**) and frontal lobe (**b**, **d**) tissues, respectively. Neuromelanin is shown in brown color and immunostaining with corresponding antibody in reddish color. Scale bars are 100 μm

**Fig. 4:**
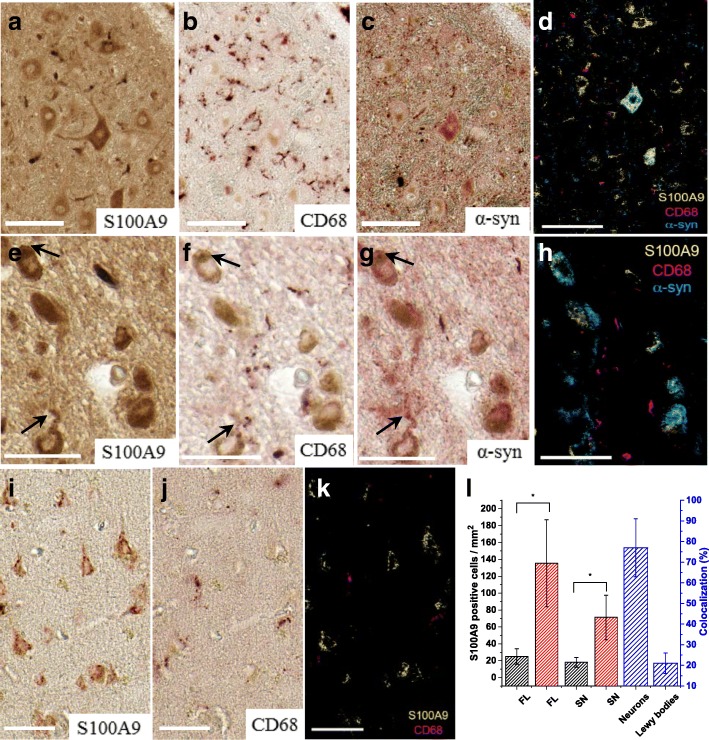
Immunohistochemical analysis of neuronal S100A9 in the PD substantia nigra and frontal lobe tissues (five cases studied). **a**–**c** Sequential immunohistochemistry of the representative PD substantia nigra region with S100A9, CD68, and α-syn antibodies, respectively. **d** Superposition of the corresponding images from **a**–**c** shown in pseudo-colors: S100A9 immunostaining is shown in yellow, α-syn in blue, and CD68 in magenta. **e**–**g** Enlarged images of the sequential immunohistochemistry of the representative substantia nigra region collected from another PD patient and conducted by using S100A9, CD68, and α-syn antibodies, respectively. Black arrows mark the accumulation of α-syn and S100A9 in small granules. **h** Superposition of the corresponding images from **e**–**g** shown in pseudo-colors: color coding as in **d**. **i**, **j** Sequential immunohistochemistry of the representative PD frontal lobe tissues with S100A9 and CD68 antibodies, respectively. **k** Superposition of the corresponding images from **i**, **j** shown in pseudo-colors: color coding as in **d**. **l** Counts of S100A9-immunopositive neuronal cells in controls are shown by black bars and in PD patients by red bars; frontal lobe is labeled by FL and substantia nigra by SN. Percentage of the co-localization of S100A9 and α-syn within neuronal cells and Lewy bodies in the substantia nigra shown by blue bars. *p* ≤ 0.05 is indicated by *. Scale bars are 100 μm in **a**–**d** and 50 μm in **e**–**k**

The substantia nigra tissue sections from five PD patients were also subjected to the sequential immunohistochemistry with pair of consecutively applied S100A9 and α-syn antibodies, which revealed that some intracytoplasmic Lewy bodies were clearly immunoreactive with both antibodies as shown in two pairs of representative images (Fig. 2a–d). The host cells, containing these Lewy bodies, displayed typical neuronal morphology (Fig. 2a–d). Both immunostaining patterns were overlapping, demonstrating the obvious co-localization of both S100A9 and α-syn within Lewy bodies. Particularly strong co-immunostaining, reflecting co-localization of these two antigens, was observed at the outer layer region, shown as a bright ring (Fig. 2c, d), though some Lewy bodies were more uniformly stained in the whole section (Fig. 2a, b). The Lewy bodies were also reactive with Congo red dye binding specifically to amyloid inclusions as shown in Fig. 2e. The Lewy bodies in the substantia nigra were also observed by using immunofluorescence, i.e., intracellular Lewy body inclusions reactive with α-syn antibodies were recognized by orange fluorescence (Fig. 2f). Some small inclusions displayed green fluorescence characteristic for S100A9-specific antibodies or yellow color, indicating the overlap of orange and green fluorescence and co-localization of both antigens (Fig. 2f). The Lewy bodies immunoreactive with α-syn antibodies were counted across all substantia nigra region in all five PD patients and their amounts were within 300–350 per case. Lewy body inclusions immunoreactive with either α-syn or S100A9 antibodies were not observed in the substantia nigra and frontal lobe tissues of control individuals (Fig. 3). We have found that ca. 20% of α-syn-immunopositive Lewy bodies in the substantia nigra were also immunopositive with S100A9 antibodies (Fig. 4l), indicating that S100A9 together with α-syn contributes to Lewy body formation. In some neuronal cells in the substantia nigra, small granular structures immunopositive with both S100A9 and α-syn antibodies were also observed (as indicated by black arrows in Fig. 4e, g), suggesting initiation of PD pathology and potential Lewy body development.

Lewy body inclusions were found also in the frontal lobe areas of all five PD patients by using both immunohistochemistry and immunofluorescence (Fig. 2g–j). They were observed as round-shaped inclusions reactive with α-syn antibodies as shown by immunohistochemistry (Fig. 2g) or orange spheres as shown by immunofluorescence (Fig. 2j). Some inclusions were rather large but irregular in shape (Fig. 2h). In some neurons, the granular structures displaying orange, green, or yellow fluorescence were noticed, indicating the deposition of α-syn, S100A9, or their co-localization, characteristic for the initiation of amyloid pathology (Fig. 2i, j). The numbers of Lewy bodies in the PD frontal lobe areas were within a wider range of 80 to 240 per case (versus 300–350 per case in the substantia nigra) and up to 16 times lower per square millimeter of the tissue than in the substantia nigra. In the frontal lobe, co-immunostaining of the Lewy bodies with α-syn and S100A9 antibodies was observed in ca. 20% cases, similar to the substantia nigra.

### Neuronal S100A9 in the PD substantia nigra and frontal lobe regions

The brain tissues of five PD patients compared to four controls were also examined for the intracellular presence of both α-syn and S100A9 antigens, which may precede the aggregation and Lewy body formation. Both proteins are known to be expressed in neuronal cells, and the elevated levels of S100A9 have been reported in Alzheimer’s disease and aging [18, 24, 26]; however, no information is available for PD-affected brain tissues. Specifically, the tissue sections from the PD-affected midbrain and frontal lobe areas have been analyzed, since both these areas were reported to be dysfunctional in PD [41]. Numerous brightly stained S100A9-immunopositive cells with a characteristic neuronal morphology were observed both in the substantia nigra and in the frontal lobe areas (Fig. 4a, e, i). Some of the neurons in the substantia nigra contain also neuromelanin, which was reflected in their characteristic brown color compared to the antigen-specific reddish-brown staining (Fig. 4f, g).

Sequential immunohistochemistry with antibodies to S100A9, α-syn, and CD68 (specific for activated microglial cells and macrophages) was performed to examine if S100A9 and α-syn are produced also by microglial cells in the substantia nigra and two representative immunostaining sequences are presented in Fig. 4a–d and Fig. 4e–h, respectively. The overlap of individual immunostaining patterns for S100A9 and α-syn in the arbitrary colors indicates the co-localization of these proteins in neuronal cells. The co-localization of the S100A9/α-syn and CD68 immunostaining patterns in the substantia nigra was at the level of immunohistochemical detection, indicating that S100A9 is accumulated primarily in neurons (Fig. 4d, h), though we cannot exclude its presence in microglial cells. In the substantia nigra, α-syn, but not S100A9, was also accumulated in Lewy neurites (Figs. 2b and 4c), which is a pathological hallmark of PD. Substantial amount of S100A9-immunopositive neurons were observed in the frontal lobe area of all five PD patients, as shown in representative image in Fig. 4i, and some neuronal cells were α-syn positive as shown by immunofluorescence (Fig. 2i, j). The sequential immunohistochemistry with S100A9 (Fig. 4i) and CD68 (Fig. 4j) antibodies, followed by the superposition of immunostaining patterns in the arbitrary colors (Fig. 4k), indicated that there is no co-localization of the staining patterns and therefore no detectable S100A9 in microglial cells in the frontal lobe tissues.

In the tissue samples from both the substantia nigra and frontal lobe of all four control individuals, intra-neuronal staining with α-syn antibodies was also observed shown in reddish-brown color (Fig. 3a, b), while brown coloring in the substantia nigra neurons corresponded to neuromelanin. However, the amount of cells immunoreactive with α-syn antibodies was much lower in the controls than in the PD patients. Since the α-syn antibody used in the study detects also the physiological form of α-syn and not selective for the disease-associated conformation, we interpret the occasional cytoplasmic staining as physiological expression of α-syn in neurons. This is clearly different from the inclusion-body pathology (i.e., Lewy bodies) seen in the PD samples.

In both brain regions of all four control individuals, the blood vessels were immunopositive for S100A9 antibodies as shown in the representative staining in Fig. 3d, indicating that S100A9 is produced there by myeloid cells. Some S100A9-immunopositive cells were also observed, but to a much lesser extent than in PD patient tissues (Fig. 3c, d).

The amounts of S100A9-immunopositive cells were counted in randomly selected areas of the substantia nigra and frontal lobe in each patient and averaged over all PD and control cases, respectively (Fig. 4l). Importantly, these numbers significantly increased in PD patients compared to controls both in the substantia nigra and frontal lobe, i.e., by ca. sixfold. Sequential immunohistochemistry with S100A9 and α-syn antibodies revealed that 77% of the neuronal cells immunopositive with α-syn antibodies in the substantia nigra were also immunopositive with S100A9 antibodies, indicating the co-localization of both antigens (Fig. 4l).

### Amyloid aggregation of α-syn and S100A9

**Fig. 5:**
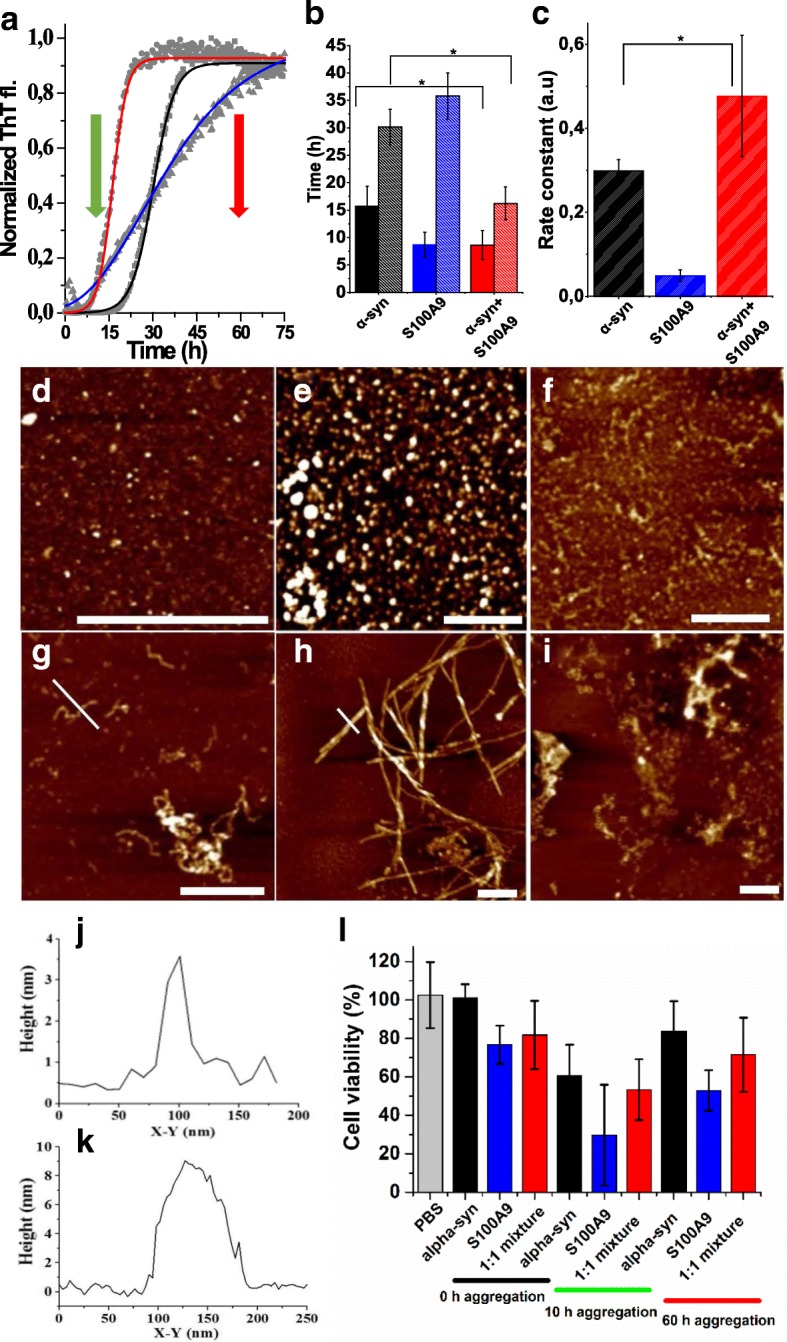
Amyloid co-aggregation and cytotoxicity of S100A9 and α-syn. **a** Normalized kinetic curves of amyloid formation monitored by ThT fluorescence and fitted by sigmoidal function for 70 μM α-syn (in black), 70 μM S100A9 (blue), and both proteins taken at equimolar ratio (red). Experimental data points are shown in gray. **b** Lag phase (dark bars) and midpoint of growth phase (light bars) of the amyloid formation kinetics derived from fitting. Protein samples are indicated under the *x*-axis and in the same color coding as in **a**. **c** Growth rate constant derived from fitting. Protein samples are indicated under the *x*-axis and in the same color coding as in **a**. Error bars represent SD. *p* ≤ 0.05 is indicated by *. **d**–**i** AFM height images of oligomeric/protofilament species of **d** S100A9, **e** α-syn, and **f** their equimolar mixture formed after 10 h incubation; amyloid fibrils of **g** S100A9, **h** α-syn, and **i** their co-aggregates at equimolar ratio of both proteins formed after 60 h incubation. Scale bars equal to 500 nm in all images. **j**, **k** AFM cross-sections of amyloid fibrils of S100A9 and α-syn, as shown by white cross-sections in **g**, **h**, respectively. **l** Viability of SH-SY5Y cells measured by WST-1 assay after 24 h co-incubation with α-syn and S100A9 species. Viability of cells treated with PBS alone is shown by gray bar; cells treated with 10 μM α-syn, 10 μM S100A9, or their equimolar mixture are shown by black, blue, and red bars, respectively. The durations of amyloid sample aggregation prior adding to cell culture, i.e., 0, 10, and 60 h, are indicated under the *x*-axis by black, green, and red bars, respectively; the oligomeric and fibrillar sample time collections are marked by red and green arrows, respectively, in the amyloid formation kinetics in **a**. Error bars represent SD of at least nine measurements. All protein samples were incubated in PBS, pH 7.4 and 37 °C

The amyloid formation kinetics of α-syn, S100A9, and their equimolar mixture, incubated under continuous agitation in PBS, pH 7.4 and 37 °C, were monitored by ThT fluorescence assay, since ThT fluorescence increases when the dye binds specifically to amyloid structures (Fig. 5a). The S100A9 fibrillation was characterized by a shorter lag phase compared to the α-syn amyloid assembly, i.e., ca. 9 vs 16 h, respectively (Fig. 5a, b). The growth phase of S100A9 fibrillation was slower than the α-syn amyloid growth with the midpoints at 36 vs 31 h, respectively (Fig. 5b). However, when both proteins were mixed at equimolar ratio, the lag phase of their joint amyloid aggregation became equal to that of S100A9 alone, while the midpoint of the growth phase reduced to ca. 16 h (Fig. 5b). The described differences in the lag times and midpoints for α-syn amyloid kinetics in the presence and absence of S100A9 were statistically significant. In addition, the growth rate constant for α-syn mixed with S100A9 was by ca. 40% faster than that of α-syn alone (Fig. 5c). The amyloid formation of α-syn was also observed in the presence of 5 and 10% S100A9 sample with preformed fibrils, characterized by smaller shift of the lag phase, growth midpoint, and apparent rate constants to lower values compared to the fibrillation of α-syn alone (Additional file 1). However, no deviations from the original fibrillation curve of S100A9 (Fig. 5a) were observed in the presence of 5 and 10% of α-syn preformed fibrils. These indicate that native S100A9, rather than its fibrils, can potentiate α-syn amyloid formation.

The amyloid formation of both proteins was monitored also by AFM imaging (Fig. 5d–i). After 10 h incubation, both S100A9 and α-syn developed round-shaped oligomers with ca. 3–4 nm and 5–6 nm heights in AFM cross-sections, respectively (Fig. 5d, e). After the same incubation time of the equimolar mixture of both proteins, the elongated protofilaments were developed coexisting together with oligomers of ca. 3–10 nm heights (Fig. 5f). After 60 h incubation, S100A9 self-assembled into curly fibrils with ca. 4 nm heights in the AFM cross-section (Fig. 5g, j), while α-syn formed mature fibrils of a few micron lengths and 8–10 nm heights in AFM cross-sections (Fig. 5h, k). Interestingly, the co-incubation for 60 h of their equimolar mixture resulted in the formation of large clustered aggregates coexisting with smaller curly and round-shaped structures (Fig. 5i).

### Cytotoxicity of S100A9 and α-syn amyloids

The cytotoxic effect on SH-SY5Y neuroblastoma cells produced by the native and amyloid species of S100A9, α-syn, and their equimolar mixture was assessed by using a WST 1 assay (Fig. 5l; statistical analysis is shown in Additional file 2). Freshly dissolved S100A9 and its mixture with α-syn added to SH-SY5Y cells caused reduction of cell viability by ca. 23 and 18%, respectively, compared to control with added PBS, while freshly dissolved α-syn did not induce statistically significant cytotoxic effect. The amyloid species of both α-syn, S100A9, and their mixture formed after 10 h incubation appeared to be the most cell toxic, causing the reduction of cell viability by ca. 40, 70, and 45%, respectively; the difference between the cytotoxicity induced by α-syn and 1:1 mixed oligomeric samples was not statistically significant. These samples were collected during the lag phase of the amyloid formation kinetics of α-syn and in the beginning of growth phase of the amyloid kinetics of S100A9 and 1:1 mixture as shown in Fig. 5a by green arrow. Therefore, the predominant amyloid species in these samples were oligomers in the α-syn and S100A9 samples and oligomers together with short protofilaments in the sample of mixed proteins as shown in the AFM images in Fig. 5d–f. Amyloid fibrils of each protein and their mixture were collected after 60 h incubation, i.e., their time point of collection is indicated by red arrow in the amyloid kinetic curves (Fig. 5a) and the amyloid structures developed in each sample during this period of time are shown in the AFM images (Fig. 5g–i). The mature amyloid fibrils of α-syn, which are shown as micron length polymers in Fig. 5h, became significantly less toxic compared to all three oligomeric samples, reducing cell viability to the level indistinguishable from control (Fig. 5l). The amyloid fibrils of mixed proteins also became significantly less toxic than S100A9 oligomers, reducing cell viability by ca. 30% compared to control. However, the change in cellular toxicity of short amyloid fibrils of S100A9 was statistically insignificant compared to those of all three oligomeric species (Fig. 5l, Additional file 2).

### Interactions of native S100A9 and α-syn

**Fig. 6:**
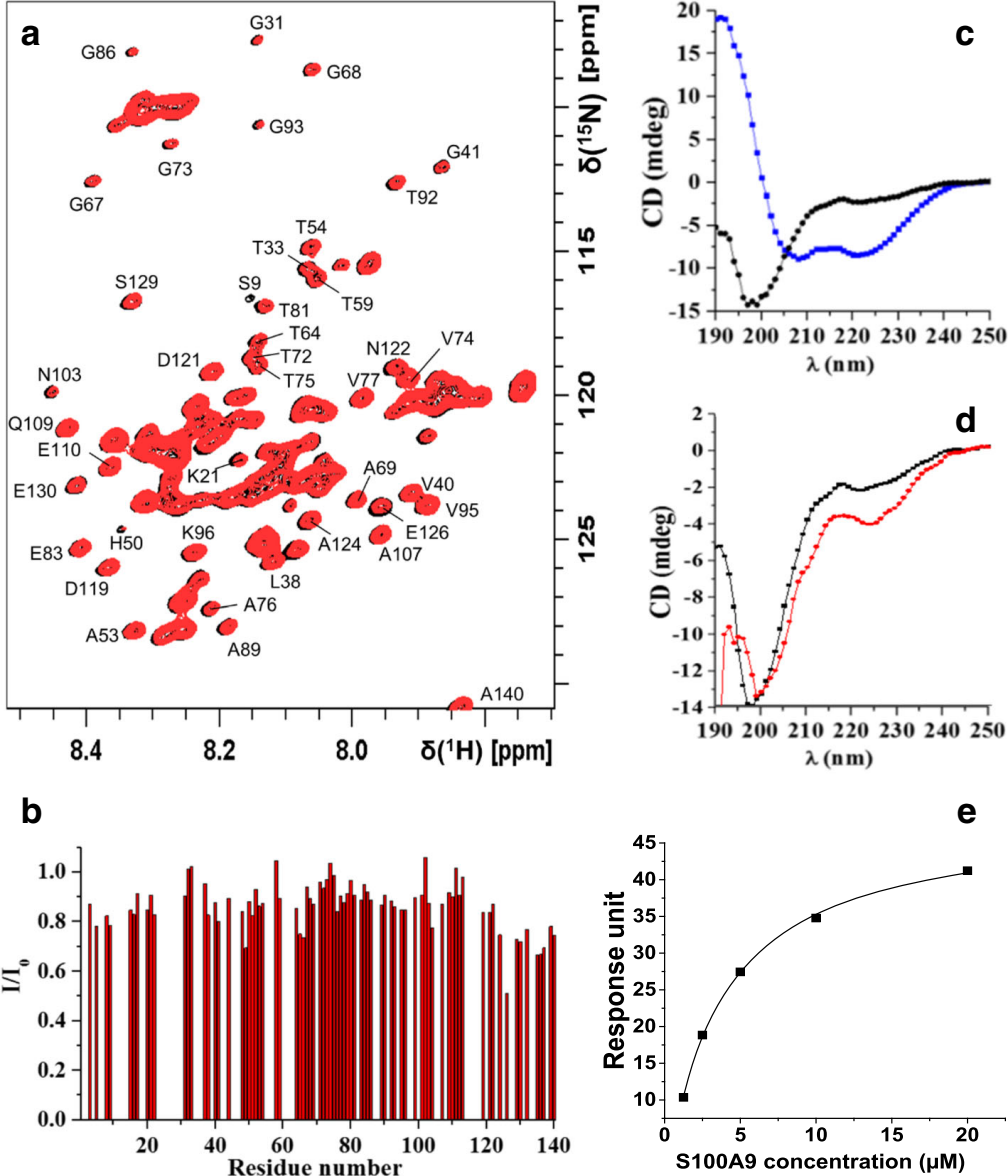
NMR, CD, and surface plasmon resonance studies of α-syn and S100A9 interactions. **a** 2D ^1^H,^15^N-HSQC spectra of 77 μM α-syn before (blue) and after (red) titration with up to 2.5 mM S100A9 in 10 mM phosphate buffer, pH 7.4 and at 10 °C. **b** Ratios of α-syn amide crosspeak intensities measured in the presence (I) and in the absence (I_0_) of S100A9, plotted as a function of α-syn amino acid residue numbers. **c** Far UV CD spectra of α-syn (black) and S100A9 (blue), 6 μM of each protein in 10 mM phosphate buffer, pH 7.4 and 20 °C. **d** Far UV CD spectrum of 6 μM α-syn alone before (black) and after addition of equimolar S100A9 (red), the signal of S100A9 was subtracted from the CD spectrum of the mixture. **e** Surface plasmon resonance response upon binding of injected S100A9 to surface-immobilized α-syn plotted as a function of S100A9 concentration

The interactions of native S100A9 and α-syn were analyzed by using solution NMR, CD spectroscopy, and surface plasmon resonance. 2D ^1^H,^15^N-HSQC spectra of 77 μM α-syn were recorded before and after addition of S100A9 performed in titration steps with up to 2.5 mM final concentration, to ensure the binding site saturation (Fig. 6a). The 2D ^1^H,^15^N-HSQC spectrum of free α-syn closely resembles previously assigned spectra of this protein under similar conditions [36], thus confirming that it exists in an essentially unstructured conformation, characterized by transiently populated α-helices in the N-terminal and central regions, but with the last 40 residues of the C-terminus being more unfolded and extended. Addition of S100A9 did not significantly perturb the positions of α-syn HSQC amide cross-peaks (Fig. 6a), but their intensities generally decreased to ca. 80% compared to the original values. Interestingly, the decrease was more pronounced in the C-terminus, i.e., to ca. 70%, suggesting a possible binding site for S100A9 in the C-terminal part of monomeric α-syn (Fig. 6b).

The CD spectrum of α-syn in the far UV region also confirmed its largely unstructured conformation, displaying the minimum of ellipticity at ca. 197 nm typical for random coil (Fig. 6c). The CD spectrum of native S100A9 is dominated by a signal corresponding to an α-helical conformation with characteristic strong minima at 220 and 208 nm (Fig. 6c). Since the α-helical structure of S100A9 is very stable, while the unfolded conformation of α-syn can be easily perturbed upon interaction with S100A9, the differential CD spectrum of their equimolar mixture was plotted, from which the contribution of the S100A9 spectrum was subtracted. The ellipticity of the minimum at 197 nm did not change in this differential spectrum, but the amplitude of the peak with a minimum at 220 nm was increased, indicating that a certain change in secondary structure is induced in α-syn upon S100A9 binding (Fig. 6d).

In order to quantify interactions between these two proteins, we have performed the titration of α-syn by increasing concentrations of S100A9 using surface plasmon resonance technique. While α-syn was immobilized on the surface of the Biacore chip, increasing concentrations of S100A9 were injected in a step-wise manner. S100A9 binding to α-syn was detected by an increase in the response signal, corresponding to the increased mass of bound material. The dissociation constant for α-syn and S100A9 interaction calculated by fitting the titration curve was ca. 5.0 ± 0.8 μM.

## Discussion

Here, we have shown that S100A9 is abundant in the PD brain tissues both intracellularly and in Lewy body amyloid deposits (Figs. 1, 2, and 4). Importantly, S100A9 possesses dual properties as a pro-inflammatory mediator and amyloidogenic protein [18, 19]. Chronic neuroinflammation is one of the hallmarks of PD pathophysiology, associated with increased pro-inflammatory mediator levels in the PD brain tissues [10–14]. Due to its inherent amyloidogenicity, S100A9 may play a more important role in PD pathology than merely being one of the disease-related pro-inflammatory mediators: it may contribute to the development of the PD amyloid-neuroinflammatory cascade in a similar way as shown previously in Alzheimer’s disease [18]. Immunohistochemical analysis showed that S100A9 is present in 20% of all α-syn-immunopositive Lewy bodies in the PD substantia nigra and frontal lobe. Within those bodies, S100A9 is clearly co-localized with α-syn either within the same outer layer (Figs. 1 and 2) or distributed throughout the whole Lewy body section (Fig. 2). Lewy bodies were evenly spread throughout the substantia nigra, which is typical for PD pathology. Lewy bodies were also observed in the frontal lobe areas of PD patients (Braak stages 4–6), though they were in smaller quantities (Fig. 2g–j). Lewy bodies were not observed anywhere in the brain tissues of the controls (Fig. 3).

AFM imaging of Lewy bodies in the substantia nigra enabled us to measure their dimensions more accurately. Lewy bodies in the substantia nigra appeared to display very regular structures, characterized by rather tight margins for their outer and inner diameters and their annuli thicknesses proportional to the diameters (Fig. 1). This signifies that Lewy bodies were formed via common mechanisms. The fact that they have a dense core, as reported previously [42], might be the underlying reason for the lack of immunostaining in their central area. The immunopositive outer layer could be indicative of the lamellar growth mechanism, which may be a diffusion-limited process for the deposited protein and depend also on the life span of the host cells, which provide the protein assembly environment.

The immunohistochemical analysis revealed that S100A9 and α-syn are present intracellularly both in the substantia nigra and frontal lobe areas of PD patients (Figs. 2 and 4). S100A9 was found to be predominantly expressed in neuronal cells, which is similar to the previous observations in the Alzheimer’s disease brain tissues [18]. However, we cannot exclude its presence in microglial cells. This indicates that the pathogenic conditions associated with oxidative stress and amyloid self-assembly in amyloid diseases such as PD and Alzheimer’s disease may induce expression of S100A9 in neurons [18]. Initially, S100A9 was discovered in myeloid cells; however, its expression under cellular stress conditions can be induced in various cell types including epithelial cells, fibroblasts as well as neurons, indicating that S100A9 may exert its functions in a plethora of cell types [43–45]. In the substantia nigra, S100A9 and α-syn were found to be co-localized in up to 77% of neuronal cells. At the same time, Lewy neurites were immunoreactive only with α-syn, but not S100A9 antibodies (Fig. 2a, b), indicating that these proteins are not always present in the same cell regions.

Perturbation of the CD spectra of α-syn in the presence of S100A9 indicated that S100A9 and α-syn interact with each other, thereby causing additional secondary structure formation of natively unfolded α-syn molecules (Fig. 6c, d). The apparent dissociation constant of their interactions is ca. 5 μM as determined in a surface plasmon resonance technique (Fig. 6e). The α-syn HSQC spectrum recorded in the presence of S100A9 shows a decrease of cross-peak intensities, particularly for residues corresponding to the C-terminal region of α-syn, suggesting the location of the S100A9 binding site (Fig. 6a, b). It has been shown previously that transient interactions between the α-syn C-terminal and N-terminal or central NAC regions are important in maintaining its natively unfolded structure, thereby preventing α-syn aggregation and fibrillation [46]. Contacts between α-syn and S100A9, that perturb such aggregation inhibiting self-interactions, could therefore enhance α-syn aggregation.

Indeed, co-aggregation of α-syn and S100A9 occurs significantly faster than amyloid formation of the individual proteins (Fig. 5a). Interestingly, native S100A9 is more prone to potentiate α-syn amyloid formation than its fibrils. Previously, similar effect of native S100A6, which is structurally homologous to S100A9, was observed on the amyloid fibrillation of superoxide dismutase-1 (SOD-1) [47]. In addition to the removal of the amyloid inhibiting interactions within α-syn primary structure, the faster nucleation process of S100A9, characterized by shorter lag phase than the lag phase of α-syn alone (Fig. 5a), may provide seeding effect on α-syn amyloid growth nuclei. Indeed, the amyloid formation of S100A9 was well described by the Finke-Watzky model of nucleation-autocatalytic growth with dominant nucleation phase, while there was no evidence of the secondary nucleation pathways initiated on S100A9 fibrillar surfaces [18, 20], which may be the reason for the small cross-seeding effect of its fibrils on α-syn fibrillation (i.e., inducing small but significant shortening of α-syn fibrillation lag phase and growth midpoint as shown in Additional file 2). At the same time, S100A9 fibrils are characterized by highly hydrophobic surfaces as shown previously [18, 20] and therefore, they may sequestrate α-syn molecules from solution, effectively reducing its concentration, which manifested in decreased α-syn fibrillation rate in their presence.

The co-aggregates of mixed S100A9 and α-syn, sampled at the oligomer and fibrillar stages, are significantly larger in size than the corresponding amyloid structures of the individual proteins (Fig. 5d–i), and this correlates with a reduction of S100A9 amyloid oligomer cytotoxicity (Fig. 5l). As the amyloid oligomers of S100A9 are more toxic than those of α-syn, S100A9 and α-syn co-aggregation effectively rescues cells from S100A9 amyloid cytotoxicity. Interestingly, similar effect has been observed previously upon the co-aggregation of S100A9 and Aβ_1-40_/Aβ_1-42_, respectively, when interactions with the corresponding polypeptides also rescued S100A9 oligomer cytotoxicity [18]. Due to the abundance of S100A9 in neuronal tissues, S100A9 oligomers may otherwise cause huge neuronal damage. This implies also that the co-aggregation of amyloidogenic polypeptides potentially could serve as a sink of dangerous amyloid species, removing them from active circulation.

The abundance and inherent amyloidogenicity of S100A9 raises the question if this protein can be a trigger of pathological amyloid cascade in PD. Indeed, S100A9 is present in the neuronal cells and Lewy bodies in the substantia nigra, the region primarily affected by disease, and in the PD frontal lobe areas, which are affected by the disease at its later stages (Figs. 1, 2, and 4). Moreover, S100A9 is present in neuronal cells and blood vessels of aged patients without PD symptoms (Fig. 3). We suggest here that sustained neuroinflammation promoting the spread of amyloidogenic S100A9 in the brain tissues can be a trigger of the amyloid cascade involving α-syn and leading to PD development [11], similar to the effect of S100A9 on Aβ aggregation in the Alzheimer’s disease [18]. S100A9 could be a common denominator in a broad variety of inflammation-dependent amyloidoses, which may develop in various tissues and organs of the human body from the brain to the aging prostate, as we have shown previously [18, 48].

## Conclusions

This is the first report on the co-aggregation of α-syn and S100A9 both ex vivo and in vitro. Co-localized and co-aggregated S100A9 and α-syn were found in Lewy bodies and neuronal cells in the PD substantia nigra and frontal lobe areas. In vitro, their co-aggregation occurred significantly faster than self-assembly of individual proteins, leading to the formation of larger amyloid aggregates and mitigating the cytotoxicity of S100A9 oligomers. The finding of S100A9 involvement in PD may open a new avenue for therapeutic interventions targeting S100A9 as a pro-inflammatory protein and preventing its amyloid self-assembly in the brain tissues.

## Additional files

Additional file 1: Cross-seeding of α-syn amyloid formation by S100A9 amyloid fibrils. (A) Normalized kinetic curves of amyloid formation monitored by ThT fluorescence and fitted by sigmoidal function for 70 μM α-syn (shown in black), in the presence of 5% (magenta) and 10% (cyan) of S100A9 fibrillar samples. Experimental data points are shown in gray. (B) Lag phase (filled bars) and midpoint of growth phase (striped bars) of the amyloid formation kinetics derived from fitting. Protein samples are indicated under *x*-axis and in the same color coding as in (A). (C) Growth rate constant derived from fitting. Protein samples are indicated under *x*-axis and in the same color coding as in (A). Error bars represent SD. *p* ≤ 0.05 is indicated by *. (PDF 104 kb)

Additional file 2: Statistical analysis of the effects of S100A9, α-syn and mixed specimens on SH-SY5Y cellular viability presented in Fig. 5l. Cell viability values were compared pair-wise by using two-sample t-test, *n* ≥ 9. NS denotes non-significant difference, * − *p* ≤ 0.05 and ** − *p* ≤ 0.01. (PDF 100 kb)

## Abbreviations

AFM: Atomic force microscopy
CD: Circular dichroism
NMR: Nuclear magnetic resonance;
PD: Parkinson’s disease
ThT: Tioflavin T
α-syn: α-Synuclein
