# Supplementary figures and images for: Co-aggregation of pro-inflammatory S100A9 with α-synuclein in Parkinson’s disease: ex vivo and in vitro studies
Source: J Neuroinflammation. 2018 Jun 4;15:172. doi: 10.1186/s12974-018-1210-9 (PMC5987543; doi:10.1186/s12974-018-1210-9)

**A**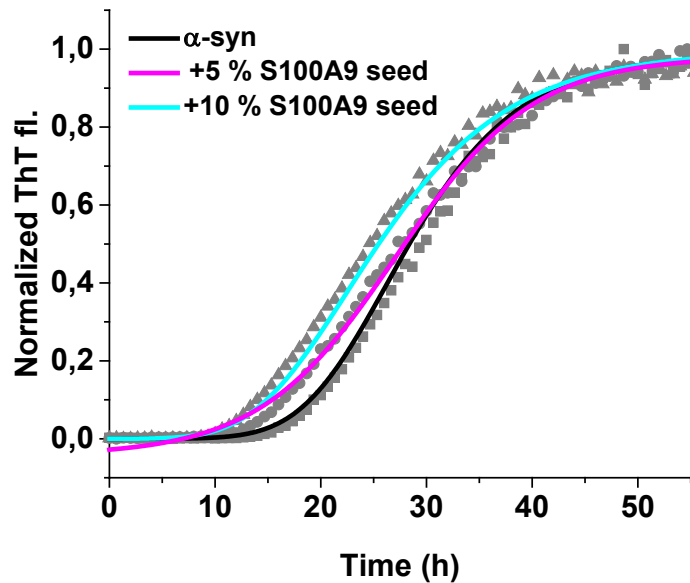**B**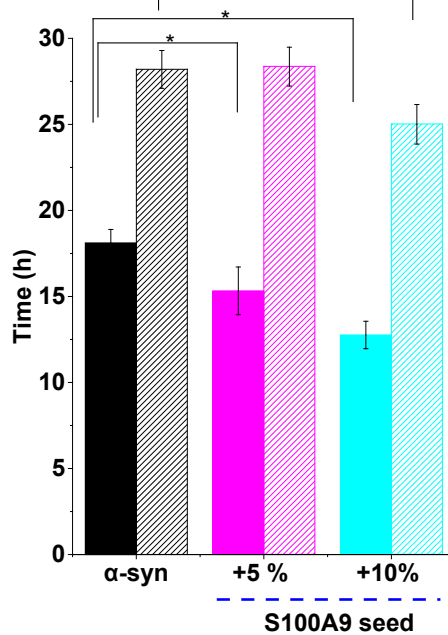**C**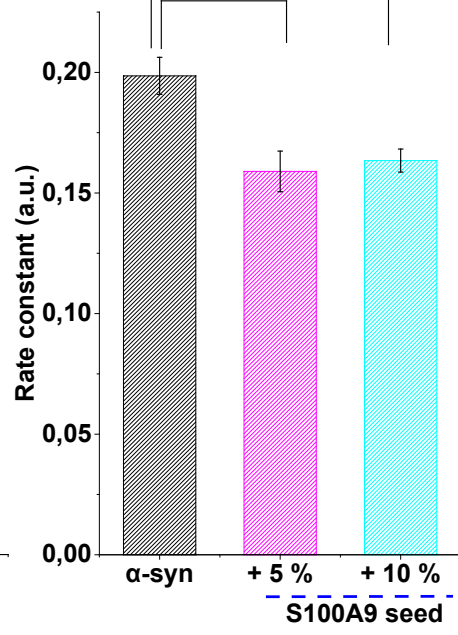

Supplement: Supplementary file 1 — Cross-seeding of α-syn amyloid formation by S100A9 amyloid fibrils. (A) Normalized kinetic curves of amyloid formation monitored by ThT fluorescence and fitted by sigmoidal function for 70 μM α-syn (shown in black), in the presence of 5% (magenta) and 10% (cyan) of S100A9 fibrillar samples. Experimental data points are shown in gray. (B) Lag phase (filled bars) and midpoint of growth phase (striped bars) of the amyloid formation kinetics derived from fitting. Protein samples are indicated under x-axis and in the same color coding as in (A). (C) Growth rate constant derived from fitting. Protein samples are indicated under x-axis and in the same color coding as in (A). Error bars represent SD. p ≤ 0.05 is indicated by *. (PDF 104 kb) [file 12974_2018_1210_MOESM1_ESM.pdf]
