# Supplementary material for: Co-aggregation of pro-inflammatory S100A9 with α-synuclein in Parkinson’s disease: ex vivo and in vitro studies
Source: J Neuroinflammation. 2018 Jun 4;15:172. doi: 10.1186/s12974-018-1210-9 (PMC5987543; doi:10.1186/s12974-018-1210-9)
Supplement: Supplementary file 2 — Statistical analysis of the effects of S100A9, α-syn and mixed specimens on SH-SY5Y cellular viability presented in Fig. 5l. Cell viability values were compared pair-wise by using two-sample t-test, n ≥ 9. NS denotes non-significant difference, * − p ≤ 0.05 and ** − p ≤ 0.01. (PDF 100 kb) [file 12974_2018_1210_MOESM2_ESM.pdf]

	Monomer α-syn	Monomer S100A9	Monomer mixture	Oligomer α-syn	Oligomer S100A9	Oligomer mixture	Fibrill α-syn	Fibrill s100a9	Fibrill mixture
Buffer	NS	*	NS	*	**	**	NS	**	*
Monomer α-syn		**	*	**	**	**	*	**	*
Monomer S100A9			NS	*	*	*	NS	*	NS
Monomer mixture				*	*	**	NS	NS	NS
Oligomer α-syn					*	NS	*	NS	NS
Oligomer S100A9						NS	**	NS	*
Oligomer mixture							*	NS	NS
Fibrill α-syn								*	NS
Fibrill S100A9									NS
